# Supplementary material for: An Oxalate Transporter Gene, AtOT, Enhances Aluminum Tolerance in Arabidopsis thaliana by Regulating Oxalate Efflux
Source: Int J Mol Sci. 2023 Feb 24;24(5):4516. doi: 10.3390/ijms24054516 (PMC10003554; doi:10.3390/ijms24054516)
Supplement: Supplementary file 1 [file ijms-24-04516-s001.zip › ijms-2071419-supplementary.pdf]

Supplementary Table S1 Primers used in the experiments.

Primer name	Primer sequence (5'-3')	Description
AtOT	F: ATGGCGGCTCCTCGG<br>R: TCATTCATATACTCTCTTTCTCT	For ORF sequence cloning
AtOT (pCAMBIA1300)	F: TCATTTGGAGAGAACACGGGGGACTATGGCGGCTCCTCGG<br>R: CCTCGCCCTTGCTCACCATGTCATTCATATACTCTCTTTC	For subcellular localizaton
AtOT (pDR196)	F: TTGGGTACCGGGCCCCCCCTCGAGGATGGCGGCTCCTCGGAATTT<br>R: CTAGTGGATCCCCCGGGCTGCAGGTCATTCATATACTCTCTTTCTCT	For yeast heterologous expression vector construction
FpOAR (pDR196)	F: GGTACCGGGCCCCCCCTCGAGGATGACCGACCTGCATCGAAG<br>R: CTAGTGGATCCCCCGGGCTGCAGGTCAGAGAAGATCTTCTTGCC	
q-*AtOT*	F: GGCGGCTCCTCGGAAT<br>R: GGCAGGCATGGTGAGGT	For qRT-PCR
q-*AtActin2*	F: TGCCAATCTACGAGGGTTTC<br>R: TTCTCGATGGAAGAGCTGGT	
atot	LP: ATGTTAAGTTCTTCAGTTTC<br>RP: TCAGAATCACCCATATTTGGTCAAA<br>LBb1.3: ATTTTGCCGATTTCGGAAC	For homozygous identification

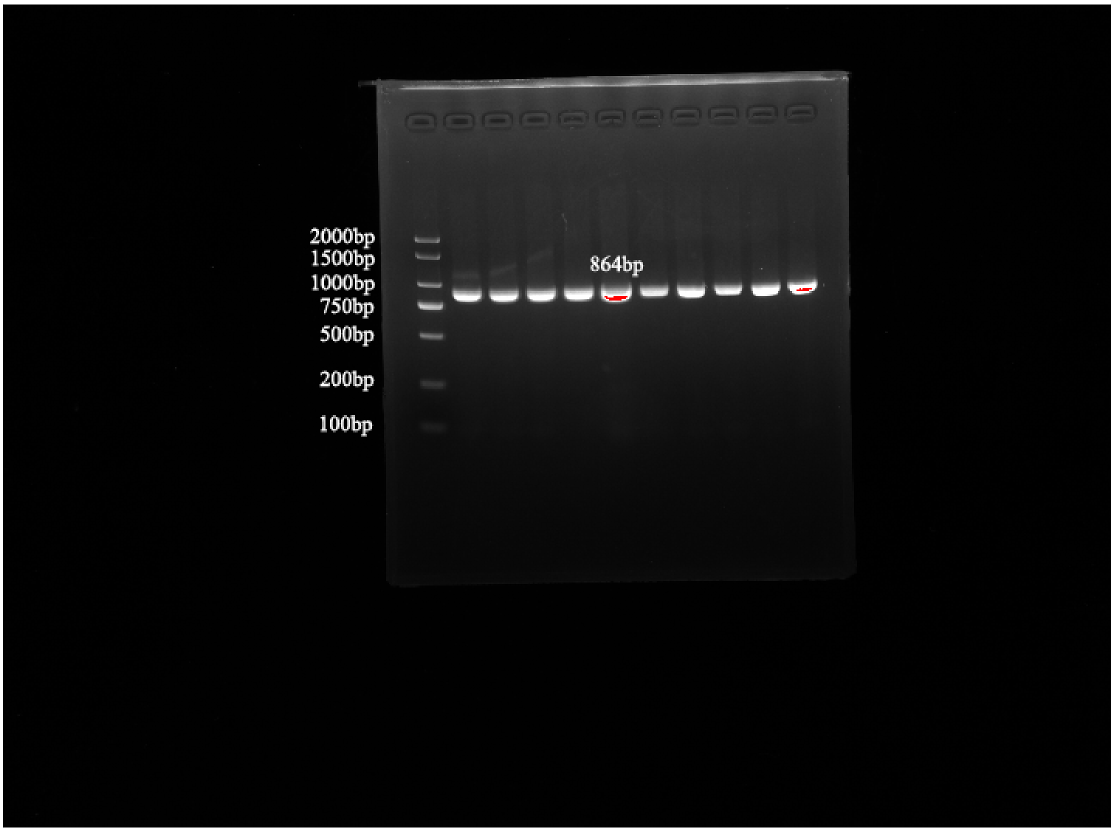

Supplementary Figure S1. The PCR amplification products of the ORF sequences of

AtOT. Maker: DL2000.

A

B

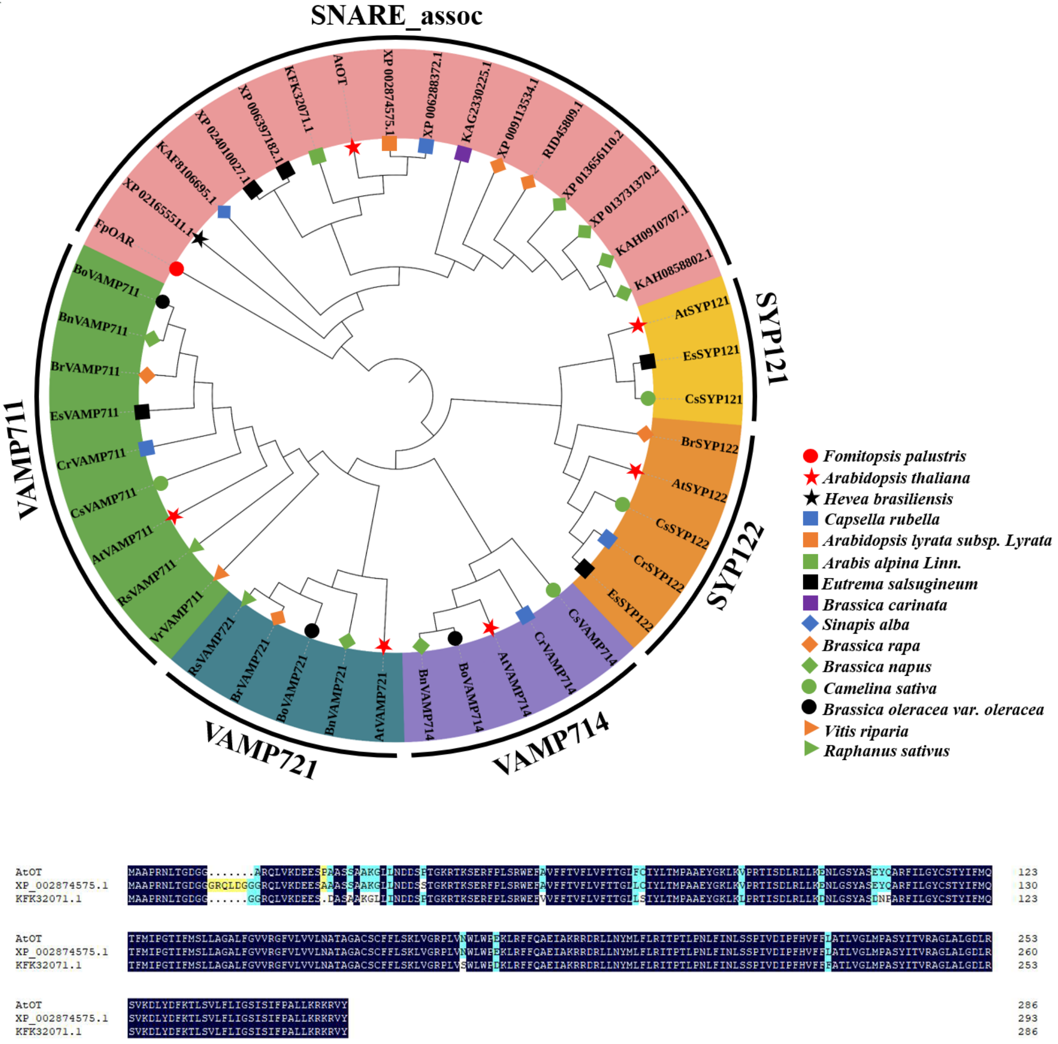

Supplementary Figure S2. Phylogenetic analysis of FpOAR, AtOT and SNARE protein members in other plants (A) and multiple sequence alignment of AtOT, XP _ 002874575.1 and KFK32071.1 (B).
